# Expression of Growth Hormone Receptors by Lymphocyte subpopulations in the Human tonsil

**DOI:** 10.1155/1998/85209

**Published:** 1998

**Authors:** Olivier Thellin, Bernard Coumans, Willy Zorzi, Ross Barnard, Georges Hennen, Ernst Heinen, Ahmed Igout

**Affiliations:** 1 Institute of Human Histology University of Liège Belgium; 2 Laboratory of Biochemistry/Endocrinology University of Liège Belgium; 3 Department of Physiology and Pharmacology University of Queensland Australia; 4 Institute of Human Histology University of Liège 20 rue de Pitteurs Liège B-4020 Belgium

**Keywords:** Human growth hormone receptor, human tonsil, hGH-N, hGH-N-R, FACS, germinal center, lymph follicle

## Abstract

The ability of human tonsillar lymphoid cells to express growth hormone receptor (hGH-N-R) was analyzed by flow cytometry. FITC-coupled recombinant human growth hormone (hGH-N) was used to reveal the receptors, in combination with phenotype markers.

Unlike T cells, tonsillar B cells constitutively express the hGH-N receptor. Quiescent cells separated from activated cells by Percoll-gradient centrifugation bear fewer receptors than activated ones. Activated T cells express hGH-N-R, but the typical germinal centre CD4^+^CD57^+^ T cells do not. These latter thus appear not to be fully activated.

Inside the lymph follicles, the germinal centre CD38^+^ B-cell population and the mantle-zone CD39^+^ B-cell population display similar levels of hGH-N-R expression, but receptor density is lower on dividing dark-zone CD38^+^CD10^+^ B cells.

Different lymphoid-cell populations thus differ markedly in their ability to express the growth hormone receptor, in relation notably to their activation status. This highlights the link between the neuroendocrine system and the active immune defense.


*Developmental Immunology*, 1998, Vol. 6, pp. 295–304
Reprints available directly from the publisher
Photocopying permitted by license only

© 1998 OPA (Overseas Publishers Association)
N.V. Published by license under the
Harwood Academic Publishers imprint,
part of the Gordon and Breach Publishing Group
Printed in Malaysia

### Expression of Growth Hormone Receptors by Lymphocyte Subpopulations in the Human Tonsil

OLIVIER THELLIN[b], BERNARD COUMANS[b], WILLY ZORZI[a], ROSS BARNARD[c], GEORGES HENNEN[b], ERNST HEINEN[a*] and AHMED IGOUT[b]

[a]*Institute of Human Histology*; [b]*Laboratory of Biochemistry/Endocrinology, University of Liège, Belgium* ; [c]*Department of Physiology and Pharmacology, University of Queensland, Australia*

(*Received 7 August 1996; In final form 9 July 1997; Accepted 10 August 1997*)

The ability of human tonsillar lymphoid cells to express growth hormone receptor (hGH-N-R) was analyzed by flow cytometry. FITC-coupled recombinant human growth hormone (hGH-N) was used to reveal the receptors, in combination with phenotype markers.

Unlike T cells, tonsillar B cells constitutively express the hGH-N receptor. Quiescent cells separated from activated cells by Percoll-gradient centrifugation bear fewer receptors than activated ones. Activated T cells express hGH-N-R, but the typical germinal centre $CD4^+CD57^+$ T cells do not. These latter thus appear not to be fully activated.

Inside the lymph follicles, the germinal centre $CD38^+$ B-cell population and the mantle-zone $CD39^+$ B-cell population display similar levels of hGH-N-R expression, but receptor density is lower on dividing dark-zone $CD38^+CD10^+$ B cells.

Different lymphoid-cell populations thus differ markedly in their ability to express the growth hormone receptor, in relation notably to their activation status. This highlights the link between the neuroendocrine system and the active immune defense.

*Keywords:* Human growth hormone receptor, human tonsil, hGH-N, hGH-N-R, FACS, germinal center, lymph follicle

### INTRODUCTION

Several studies have revealed two-way connections between the immune and the neuroendocrine systems (Mocchegiani et al., 1990; Murphy et al., 1992; Blalock, 1994; Clarke and Kendall, 1994; Mathison et al., 1994; Ottaway and Hurband, 1994; Fabris et al., 1995; Hooghe-Peters and Hooghe, 1995; Hooghe et al., 1996). This highly regulated ensemble forms a network of physiological reactions. Growth hormone produced by the pituitary, under the control of the hypothalamus, is one of the main actors in this network.

Growth hormone (hGH-N), in addition to its well-known metabolic effects, can modulate the immune system through its direct action on lymphocytes and their accessory cells. It is well established that human lymphoid cells express the growth hormone receptor

*Corresponding author. Present address: Institute of Human Histology, University of Liège, 20 rue de Pitteurs, B-4020 Liège, Belgium.

(hGH-N-R) and even produce hGH-N (Kiess and Butenandt, 1985; Badolato et al., 1994; see for review Hooghe et al., 1996). The studies establishing these facts focused solely on crude lymphoid-cell populations, taking no account of the activation or maturation levels of subpopulations present.

Here we have examined expression of hGH-N-R by lymphocyte subpopulations freshly prepared from human tonsillar lymph follicles and by a mitogen-activated lymphocyte population. We have addressed the question: Does hGH-N-R expression by lymphocytes correlate with their activation or maturation status?

Different lymphocyte subpopulations were analyzed by flow cytometry. Mantle-zone B cells were studied by CD39 labeling, germinal center B cells by CD38 labeling, and dividing germinal center B cells by $CD38^+CD10^+$ labeling (Gregory et al., 1987; Liu et al., 1992; Hardie et al., 1993; Lagresle et al., 1993; MacLennan, 1994; Kremmidiotis and Zola, 1995; Lagresle et al, 1995; Dono et al., 1996). A peculiar population of T cells has been found inside the germinal centers; these cells are $CD4^+$, most of them are $CD57^+$ (Velardi et al., 1986; Bouzahzah et al., 1996a). On the basis of CD57 expression, we purified them by MACS and analyzed their hGH-N-R expression. Activated cells were separated from quiescent ones by Percoll-gradient centrifugation.

Expression of hGH-N-R was revealed by two different means: with mAb263 (mouse anti-hGH-N-R) and with FITC-labeled hGH-N. Since both methods gave similar results (to be published), we present here the results obtained with hGH-N/FITC.

By double labeling (anti-CD/PE and hGH-N/FITC), we identified cell types and checked their hGH-N-R expression.

### MATERIAL AND METHODS

### Antibodies

Phycoerythrin (PE)-conjugated anti-CD3, anti-CD10, anti-CD19, and unconjugated anti-CD19 and anti-CD8 monoclonal antibodies were provided by DAKO (Denmark). Simultest anti-CD3/FITC/CD19/PE, anti-CD38/PE, and biotinylated anti-CD57 mAbs were purchased from Becton-Dickinson (Rutherford, NJ). Rabbit anti-human Ig coupled to polyacrylamide beads was produced by Biorad Laboratories (Richmond, VA) and used at 1/600 final dilution. Anti-CD39/PE mAb was provided by Pharmingen (USA) and streptavidin/PE by Boehringer Mannheim (Germany). OKT3 and OKT11 mAbs were kindly provided by Thierry de France (Lyon, France) and mAb263 was a gracious gift from Ross Barnard (Queensland, Australia).

### Isolation of T Cells

Tonsils from 1 to 8-year-old children were surgically removed and carried to the laboratory at 4°C in a physiological solution containing 0.4% BSA (Sigma, USA). Lymphocyte populations were prepared by gently teasing the tonsils. Free cells were harvested and T cells were separated by rosetting using sheep red blood cells (SRBC), treated with 2-aminoethylthiouronium, on a Ficoll Paque gradient (Pharmacia, Sweden). $CD3^+$ cells were selected by eliminating $CD19^+$ cells from the rosetted population (Bouzahzah et al., 1996b). For this, the cells were incubated with mouse anti-CD19 antibodies before rinsing and adding Dynabeads-coupled anti-mouse IgG (Dynal, Norway). Labeled B cells were eliminated in a magnetic field. The purity of the T cells usually exceeded 95%, as determined by cytofluorometry (FACScan, Becton-Dickinson, USA).

$CD4^+$ lymphocytes were purified similarly with anti-CD19 and anti-CD8 mAbs and Dynabeads.

$CD4^+CD57^+$ T cells were prepared (Bouzahzah et al., 1995) by means of a magnetic cell sorter (MACS, Becton-Dickinson, USA): Briefly, $CD4^+$ cells were incubated for 30 min at 4°C with anti-CD57 mAb, then rinsed and placed in contact with PE-conjugated avidin and biotinylated magnetic beads (Sanvertec, The Netherlands). The cells were transferred to a separation column in the magnetic field of a MACS apparatus. $CD4^+CD57^-$ cells passed through the column, $CD57^+$ cells were retained by the magnetic field. The resulting $CD57^+$ and $CD57^-$ cells preparations were respectively 90% and 95% pure.

### Isolation of B Cells

Of the nonrosetted cells, around 90% were B cells ($CD19^+$). The concentration of B cells was improved to 95% and 98% of total population by eliminating the contaminating T cells with OKT3 and OKT11 and then Dynabeads (Bouzahzah et al., 1995).

### Percoll Gradients

Tonsillar cells were separated (Cleary et al., 1995) by density centrifugation into high- and low-density cells on a discontinuous 30/50/100 % Percoll (Pharmacia, Sweden) gradient (1100 × g; 12 min). High-density cells were collected at the 50/100 % interface, and low-density cells at the 30/50 % interface. To preserve isotonicity (1 × PBS), the Percoll was diluted in PBS. Thus, 100% Percoll is defined as commercially available pure Percoll diluted in 0.1 volume of 10 × PBS, 50% Percoll is a 1/1 dilution of Percoll in 1 × PBS, and 30% Percoll is obtained by mixing 0.6 volume of Percoll with 1.4 volume of 1 × PBS.

### Preparation of hGH-N/FITC

Recombinant human GH-N (Kabi, Sweden) was coupled (Badolato et al., 1994; Rapaport et al., 1995) to FITC (Sigma). This was done as follow: hGH-N (1 mg/ml) was dissolved in 0.1 M sodium bicarbonate buffer, pH 9-9.4. FITC (1 mg/ml) was dissolved in dimethylsulfoxyde (DMSO, Sigma). A 250-$\mu$l FITC solution was added to the dissolved hGH-N in 30-$\mu$l aliquots dispensed at 15-sec intervals under constant stirring. The mixture was then incubated overnight in darkness at 4°C. Free FITC was removed by three rounds of dialysis against 1000 volume of sodium bicarbonate buffer solution. This was performed at 4°C in the dark.

The protein concentration and the fluorescein/protein ratio (F/P) were determined by optical densitometry at 280 nm (protein) and 492 nm (FITC).

Bovine serum albumin (1 mg/ml BSA, Sigma) was conjugated to FITC by the same protocol and used as a negative control.

### Fluorescein Labeling

Binding of hGH-N/FITC was evaluated by incubating $10^6$ lymphocytes with 5 $\mu$g hGH-N/FITC or BSA/FITC in PBS, pH 7.4, for 1 hr at 4°C in the dark. BSA/FITC was used as a negative control. After 30 min, PE-conjugated mAbs (anti-CD3, -CD10, -CD19, -CD38, or -CD39) were added at the adequate dilution. The cells were then washed twice in 1 ml cold PBS, resuspended in 750 $\mu$l cold PBS, and analyzed with the FACScan.

### RESULTS

### Saturation Curve

To test the capacity of hGH-N/FITC to bind hGH-N receptors of living cells, we prepared tonsillar lymphoid cells, labeled them with anti-CD19/PE to stain B cells, and incubated them with increasing amounts of hGH-N/FITC before measuring the percentage of FITC-positive cells with the FACS. The results indicate that saturation level begins at approximately 5 $\mu$g/ml (Figure 1). BSA/FITC was used as a negative control (Rapaport et al., 1995). The binding specificity of hGH-N/FITC has previously been proven by blocking with unlabeled hGH (Igout et al., 1995). The subsequent experiments were performed with 5 $\mu$g hGH-N/FITC/ml, which enabled us to label a high percentage of hGHN-R while limiting non-specific binding.

### Total B- and T-Cell Populations

Crude and cultured lymph-cell populations were double-labelled with hGH/N-FITC and anti-CD3/PE (for T lymphocytes) or anti-CD19/PE (for B lymphocytes) mAbs. The results (Table I and Figures 2 and 3) obtained from six tonsils show that practically no T cell express hGHN-R wherease 60-95% of B cells express it. These percentages are underestimated,

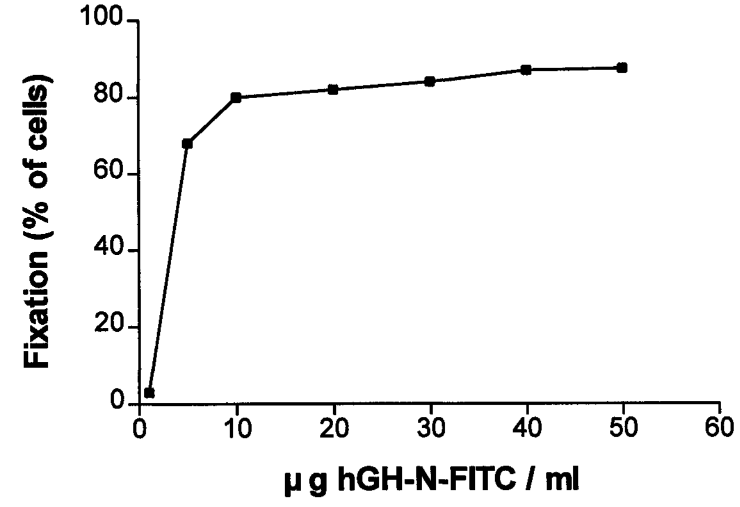

FIGURE 1 Saturation curve; ordinate: percentages of cells having bound hGH-N/FITC; abscissa: concentration of hGH-N/FITC.

since we used a nonsaturating concentration of hGH-N/FITC to stain the cells. Furthermore, experiments showed a strong rise in hGHN-R expression by T cells when these cells were activated by culturing for 24 or 48 hours in DMEM/F-12 with phycohemagglutinin L (PHA-L; 2 $\mu$g/ml); hGHN-R expression also increased, but to a lesser extent, in lipopolysaccharide-activated B cells (10 $\mu$g/ml lipopolysaccharide; same culture conditions) (data not shown). In the tonsil, most T cells thus appear not to express hGHN-R, whereas nearly all B cells exhibit a basal expression level. To verify this pattern of hGHN-R expression, we prepared and studied isolated tonsillar B- and T-cell subpopulations.

TABLE I Expression of hGHN-R by of Tonsillar B and T lymphocytes (%)

| Tonsil no. | $CD3^+$ cells | $CD19^+$ cells |
|---|---|---|
| 1 | <5 | 67 |
| 2 (rosetted cells) | <5 | 63 |
| 3 | <5% | 82 |
| 4 | ND | 77 |
| 5 | ND | 76 |
| 6 | <5 | 95 |

### T-Cell Subpopulations

We double-labeled subpopulations of T cells ($CD4^+$, $CD8^+$, or $CD57^+$) in mixed B-, T-cell populations and purified $CD3^+$ or $CD4^+CD57^+$ cells with hGH-N/FITC. The results (Table II and Figure 2) show that in all tonsillar T-cell subpopulations, even for the $CD57^+$ subpopulation, less than 5% of the cell express hGHN-R. No difference was seen between the $CD4^+$ and $CD8^+$ populations. Incubation of these cells with PHA-L enhanced their hGH-N-R expression (data to be published).

### B-Cell Subpopulations

Crude B-, T-cell populations or purified $CD19^+$ B cells were double-labeled with hGH-N/FITC and with anti-CD10, anti-CD19, anti-CD38, or anti-CD39/PE mAbs (Figure 4). The Mann-Whitney test was not applicable to either preparations because the results varied considerably from tonsil to tonsil (six tonsils, $\alpha = 5\%$) (Table III and Figure 3). To analyze differences between cell subpopulations, we thus examined each tonsil separately. In all six tonsils, $CD38^+$ and $CD39^+$ cells were found systematically to express more hGHN-R per cell than $CD38^+CD10^+$ cells. Since this is probably due to the proliferation status of

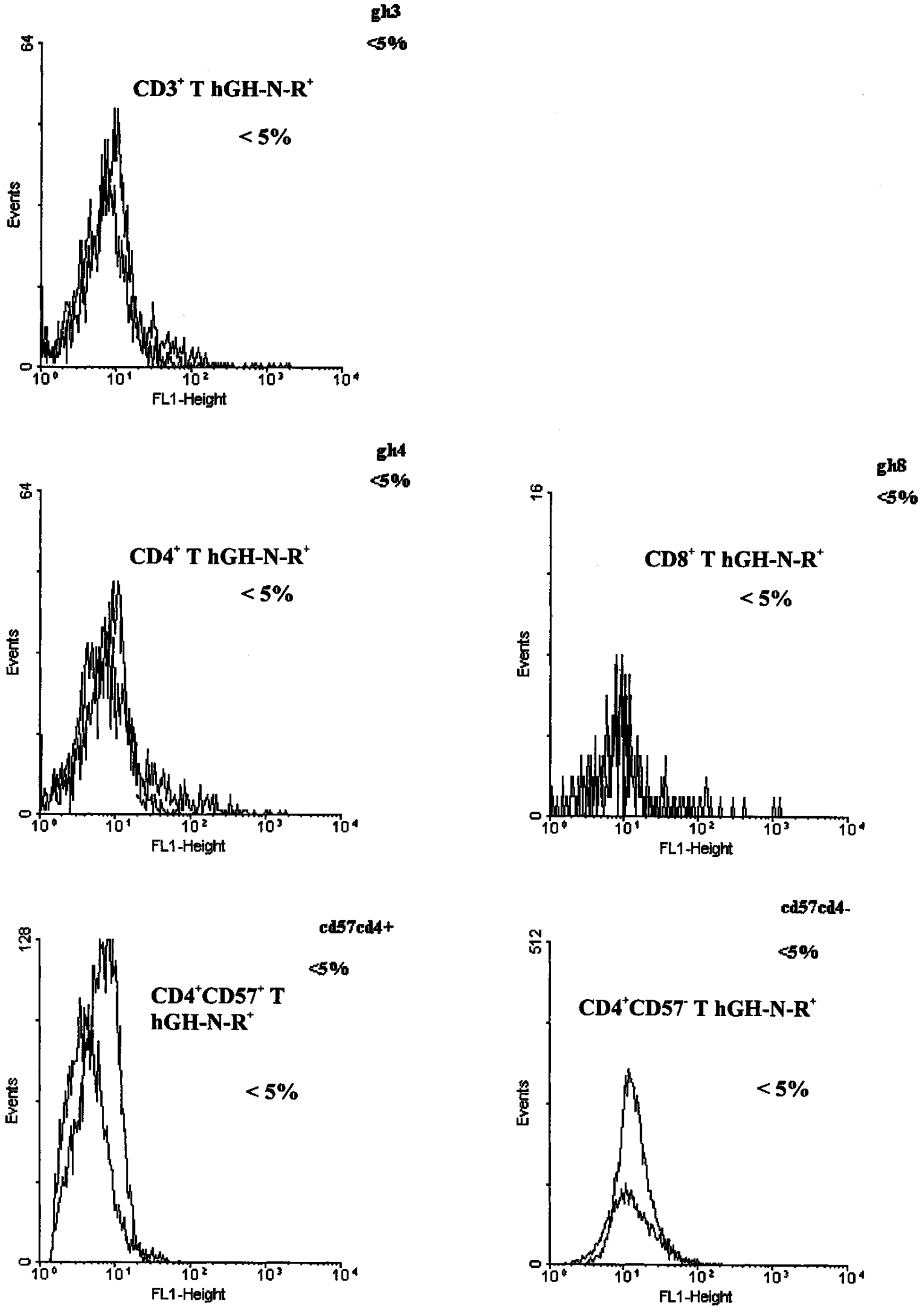

FIGURE 2 Percentages of T-cell subpopulations expressing hGH-N-R.

CD38+CD10+ cells, we investigated hGH-N-R expression among high-density (nonactivated) and low-density (activated) tonsillar B and T lymphoid cells, separated on Percoll gradients.

### T-Cell Gradients

Unseparated lymph cells were centrifuged on Percoll gradients, yielding high-density (small, nonactivated) and low-density (large, activated) cells. Both populations were investigated by double-labeling with hGH-N/FITC and anti-CD3/PE, anti-CD4/PE, or anti-CD8/PE mAbs. The results (Table IV), highly variable between tonsils, constantly show that more low-density T cells express hGHN-R than high-density T cells. The percentage of hGH-N-R positive cells is lower, nevertheless, among activated (low-density) T cells than in the corresponding low-density B-cell population.

### B-Cell Gradients

Unseparated lymph cells were centrifuged on Percoll gradients, yielding high-density (small, nonactivated) and low-density (large, activated) cells. Both populations were investigated by double-labeling with hGH-

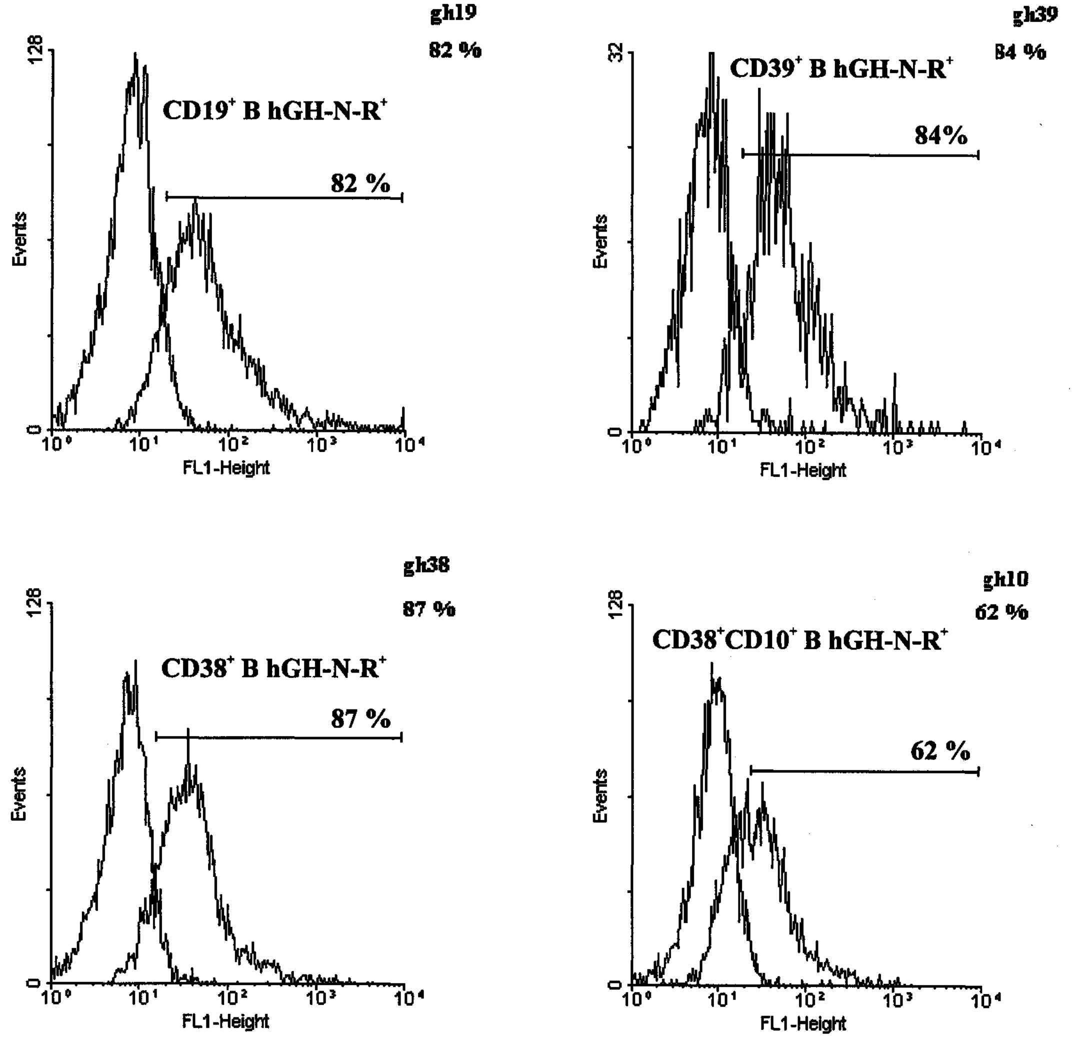

FIGURE 3 Percentages of B-cell subpopulations expressing hGH-N-R.

TABLE II Expression of hGH-N-R by Tonsillar T-Cell Subpopulations (%)

| Tonsil no. | $CD4^+$ cells | $CD8^+$ cells | $CD4^+CD57^-$ cells | $CD4^+CD57^+$ cells |
|---|---|---|---|---|
| 1 (rosetted cells) | ND | ND | <5 | <5 |
| 2 | ND | ND | <5 | <5 |
| 3 | <5 | <5 | ND | ND |
| 4 | <5 | <5 | ND | ND |
| 5 | ND | ND | <5 | <5 |

N/FITC and anti-CD10/PE, anti-CD19/PE, anti-CD38/PE, or anti-CD39/PE mAbs. The results (Table V and Figure 5) show that low-density B cells express slightly more hGHN-R than high-density B cells and also more than the total B-cell population prior to separation. The hGH-N-R density varies on low-density B cells according to cell type: $CD10^+$ cells express less hGHN-R than other cell types. Here again, high variability between tonsils is apparent, but the results for each tonsil show a higher percentage of labeled cells in the low-density population. In both the high- and low-density populations, fewer $CD38^+CD10^+$ cells than $CD38^+CD10^-$ or $CD39^+$ cell express the receptor.

### DISCUSSION

Germinal centers develop in the secondary follicles of lymphoid organs by B-cell proliferation during a T-cell-dependent antibody response (MacLennan et al., 1992; MacLennan, 1994). Each germinal center is divided into a dark and a light zone. Centroblasts proliferate in the dark zone, and, migrating to the light zone, mature in contact with antigen-presenting follicular dendritic cells (FDC). During their maturation, $CD38^+CD10^+$ B cells first lose CD10, then CD38 at the end of maturation (Kremmidiotis and Zola, 1995), and finally $CD39^+$ after maturation has finished (Lagresle et al., 1993).

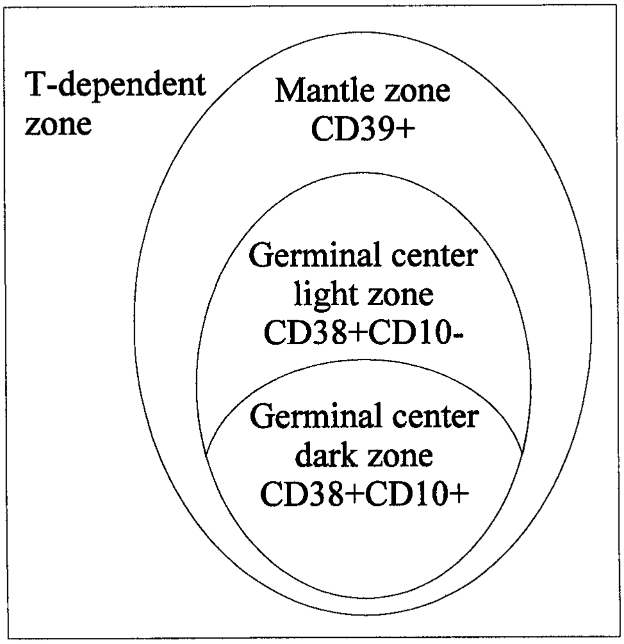

FIGURE 4 Schematic representation of a secondary lymph follicle in relation to a B-cell phenotype.

TABLE III Expression of hGH-N-R by tonsillar B cell subpopulations (%)

| Tonsil no. | $CD38^+$ cells | $CD38^+CD10^+$ cells | $CD39^+$ cells |
|---|---|---|---|
| 1 | 64 | 59 | 72 |
| 2 (rosetted cells) | 72 | 63 | 65 |
| 3 | 87 | 62 | 84 |
| 4 | 86 | 68 | 75 |
| 5 | ND | 70 | ND |
| 6 | ND | 87 | 89 |

TABLE IV Expression of hGH-N-R by Quiescent (High-Density) or Activated (Low-Density) T Cells

| Tonsil no. | High-density T cells | | | Low-density T cells | | |
|---|---|---|---|---|---|---|
| | $CD3^+$ | $CD4^+$ | $CD8^+$ | $CD3^+$ | $CD4^+$ | $CD8^+$ |
| 1 | <5 | ND | ND | 12 | ND | ND |
| 2 | <5 | <5 | <5 | 69 | 75 | 75 |
| 3 | <5 | <5 | <5 | 25 | 22 | 26 |

TABLE V Expression of hGH-N-R by Quiescent (High-Density) or Activated (Low-Density) B Cells (%)

| Tonsil no. | High-density B cells | | | | Low-density B cells | | | |
|---|---|---|---|---|---|---|---|---|
| | $CD19^+$ | $CD10^+$ | $CD38^+$ | $CD39^+$ | $CD19^+$ | $CD10^+$ | $CD38^+$ | $CD39^+$ |
| 1 | 70 | 55 | 60 | 70 | 83 | 81 | 83 | 82 |
| 2 | 73 | 53 | 60 | 57 | 82 | 76 | 80 | 80 |
| 3 | 92 | 90 | 85 | 85 | 95 | 92 | 97 | 95 |
| 4 | 91 | 89 | 93 | 92 | 95 | 91 | 97 | 96 |

Kiess and Butenandt (1985) have detected hGHN-R on mononuclear cells from peripheral venous blood. Here we have investigated the capacity of subpopulations of tonsillar B and T lymphocytes to express hGHN-R. Using fluorescein-labeled human growth hormone, cytometry, and cell-selection procedures, we have obtained new data on hGHN-R. Unlike tonsillar T cells, tonsillar B cells constitutively express these receptors. T cells do so transiently when activated. Interestingly, the peculiar $CD4^+CD57^+$ germinal center T cells do not express hGH-N-R. This confirms data from other authors, indicating that these cells are only in a preactivated state (Bouzahzah et al., 1995).

Among the follicular B cells, the dark-zone $CD38^+CD10^+$ B cells possess few hGH-N-R. Perhaps proliferation causes B cells to reduce the density of hGH-N-R on their surface: During proliferation, activated cells express less hGH-N-R.

These differences between B and T cells and between resting and proliferating cells were observed on both mixed (crude) and purified lymphoid-cell populations and after centrifugation in Percoll. The results are confirmed by *in vitro* studies on mitogen-activated lymphoid cells (in preparation). Contamination by other cells bearing the CD of interest does not appear significant, since most of our data were obtained on highly purified B- or T-lymphocyte populations.

Since lymphocyte activation requires antigen recognition and costimulatory signals given by accessory or lymphoid cells, we can now hypothesize that activated cells bear higher numbers of certain receptors rendering them particularly receptive to messages, such as growth hormone, from the neuroendocrine system. Quiescent B cells appear more sensitive to growth hormone signaling than T cells, but activation opens the latter to the influence of growth

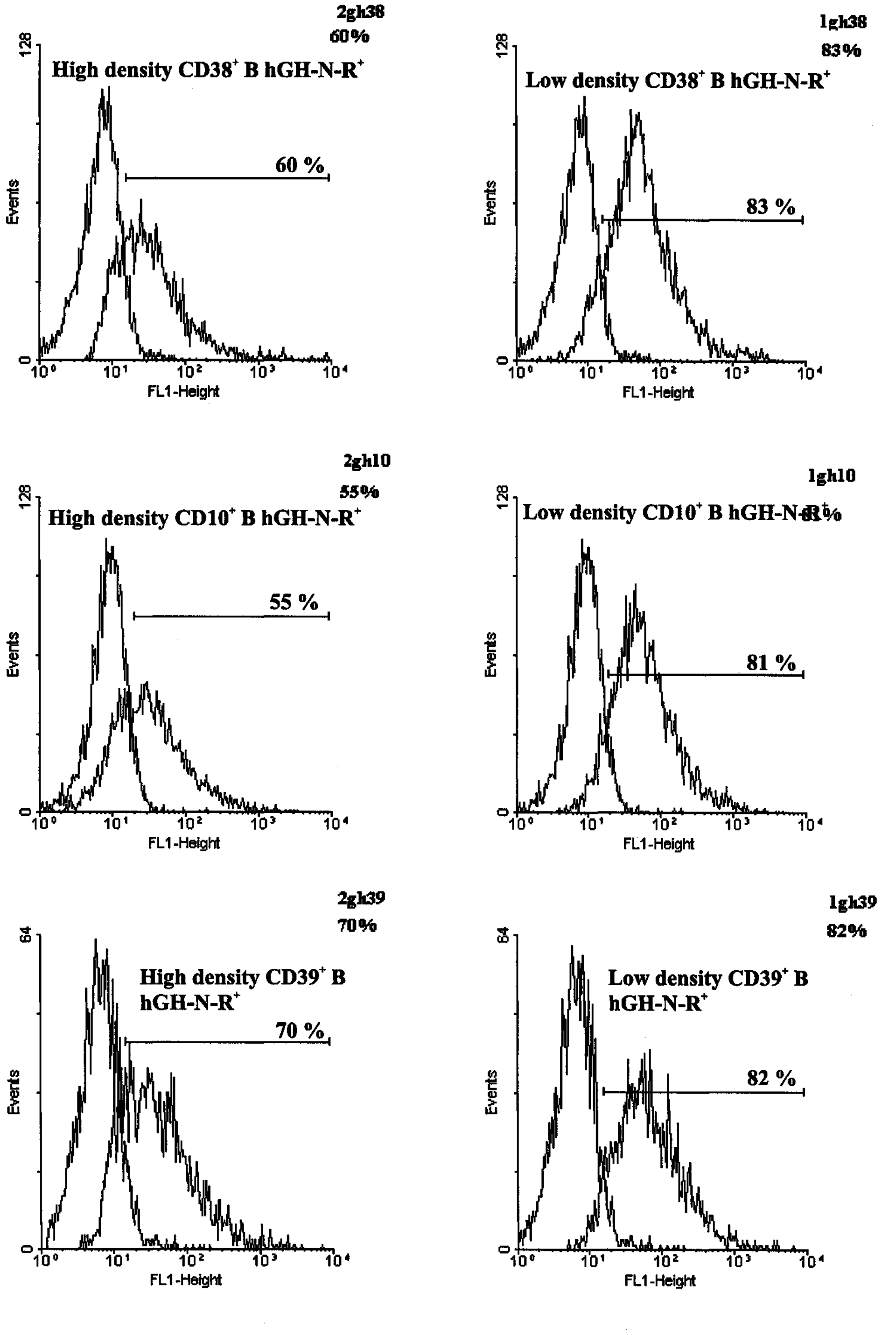

FIGURE 5 Percentages of high- and low-density B-cell subpopulations expressing hGH-N-R.

hormone. The sensitivity of the immune system to signals of the neuroendocrine system thus depends on its stimulation level.

These observations reinforce the view that hGH-N is part of the immune system regulation machinery.

### Acknowledgments

The authors thank M. Jackers and A. M. Greimers for excellent technical assistance. This work was supported by the Convention Région Wallonne, Université de Liège 2640.

### References

Badolato R., Bond H.M., Valerio G., Petrella A., Moorone G., Aters M.J., Venuta S. and Tenore A. (1994). Differential expression of surface membrane GH receptor on human PBL detected by dual fluorochrome flow cytometry. J. Clin Endocrinol. Metab. **79**(4):984-990.

Blalock J.E. (1994). The syntax of immune-neuroendocrine communication. Immunol. Today **15**(11):504-511.

Bouzahzah F., Antoine N., Simar L.J. and Heinen E. (1996b). Chemotaxis-promoting and adhesion properties of human tonsillar FDC clusters. Dev. Immunol. **147**:165-173

Bouzahzah F., Bosseloir A., Heinen E. and Simar L.J. (1995). The human CG CD4+ CD57+ T cells act differently on B cells than do classical T helper cells. Devel. Immunol. **4**:189-197.

Bouzahzah F., Suzuki S., Bosseloir A., Simar L.J. and Heinen E. (1996a). T cells in contact with follicular dendritic cells. In Follicular Dendritic Cells in Normal and Pathological Conditions, Heinen, E., ed. R.G. Landes), pp. 79-96.

Clarke A.G. and Kendall M.D. (1994). The thymus in pregnancy: The interplay of neural, endocrine and immune influences. Immunol. Today **15**(11):545-551.

Cleary A.M., Fortune S.M., Yellin M.J., Chess L. and Lederman S. (1995). Opposing roles of CD95 (FAS/APO-1) and CD40 in the death and rescue of human low density tonsilar B cells. J. Immunol. **155**:3329-3337.

Dono M., Burgio V.L., Tacchetti C., Favre A., Augliera A., Zupo S., Taborelli G., Chiorazzi N., Grossi C.E. and Ferrarini M. (1996). Subepithelial B cells in the human palatine tonsil. I. Morphologic, cytochemical and phenotypic characterization. Eur. J. Immunol. **26**:2035-2042.

Fabris N., Mocchegiani E. and Provinciali M. (1995). Pituitary-thyroid axis and immune system: A reciprocal neuroendocrine-immune interaction. Horm. Res. **43**:29-38.

Gregory C.D., Tursz T., Edwards C.F., Tetaud C., Talbot M., Caillou B., Rickinson A.B. and Lipinski M. (1987). Identification of a subset of normal B cells with a Burkitt's lymphoma (BL)-like phenotype. J. Immunol. **139**:313-318.

Hardie D.L., Johnson G.D., Khan M. and MacLennan I.C.M. (1993). Quantitative analysis of molecules which distinguish functional compartments within germinal centers. Eur. J. Immunol. **23**:997-1004.

Hooghe R., Kooijman R. and Hooghe-Peters E.L. (1996). Growth hormone and insulin-like growth factor I are haemopoietic and lymphoid growth and differentiation factors. In The Complexity of Endocrine systems, Ranke M. ed. (Mannheim), pp. 45-69.

Hooghe-Peters E.L. and Hooghe R. (1995). Growth Hormone, Prolactin and IGF-I as Lymphohemopoietic Cytokines (Austin, TX: R.G. Landes).

Igout A., Frankenne F., L'Hermite-Baleriaux M., Martin A. and Hennen G. (1995). Somatogenic and lactogenic activity of the recombinant 22 kDa isoform of human placental growth hormone. Growth Reg. **5**(1):60-65.

Kiess W. and Butenandt O. (1985). Specific growth hormone receptors on human peripheral mononuclear cells: Reexpression, identification and characterization. JCEM **60**(4):740-746.

Kremmidiotis G. and Zola H. (1995). Changes in CD44 expression during B cell differentiation in the human tonsil. Cell. Immunol. **161**:147-157.

Lagresle C., Bella C., Daniel P.T., Krammer P.H. and Defrance T. (1995). Regulation of germinal center B cell differentiation. J. Immunol. **154**:5746-5756.

Lagresle C., Bella C. and Defrance T. (1993). Phenotypic and functional heterogeneity of the $IgD^-$ B cell compartment: Identification of two major tonsillar B cell subsets. Int. Immunol. **5**(10):1259-1268.

Liu Y.J., Johnson G.D., Gordon J. and MacLennan C.M. (1992). Germinal centers in T-cell-dependent antibody responses. Immunol. Today **13**:17-21.

MacLennan I.C.M. (1994). Germinal centers. Annu. Rev. Immunol. **12**:117-139.

MacLennan I.C.M., Liu Y.J. and Johnson G.D. (1992). Maturation and dispersal of B-cell clones during T cell-dependent antibody responses. Immunol. Rev. **126**:143-161.

Mathison R., Davison J.S. and Befus A.D. (1994). Neuroendocrine regulation of inflammation and tissue repair by submandibular gland factor. Immunol. Today **15**(11):527-532.

Mocchegiani E., Paolucci P., Balsamo A., Cacciari E. and Fabris N. (1990). Influence of growth hormone on thymic endocrine activity in humans. Horm. Res. **33**:248-255.

Murphy W.J., Durum S.K. and Longo D.L. (1992). Role of neuroendocrine hormones in murine T cell development Growth hormone exerts thymopoietic effects in vivo. J. Immunol. **149**(12):3851-3857.

Ottaway C.A. and Hurband A.J. (1994). The influence of neuroendocrine pathways on lymphocyte migration. Immunol. Today **15**(11):511-517.

Rapaport R., Sills I.N., Green L., Barrett P., Labus J., Skuza K.A., Chartoff A., Goode L., Stene M. and Petersen B.H. (1995). Detection of human GH receptors on IM9 cells and peripheral blood mononuclear cell subsets by flow cytometry: Correlation with GH-BP levels. J. Clin. Endocrinol. Metab. **80**(9):2612-2619.

Velardi A., Tilden A.B., Millo R. and Grossi C.E. (1986). Isolation and characterization of Leu7+ germinal-center cells with the helper cell phenotype and granular lymphocyte morphology. J. Clin. Immunol. **6**:205-215.

